# Preferred J-pop music and visual memory retrieval: an exploratory pilot fMRI study

**DOI:** 10.3389/fnhum.2026.1775292

**Published:** 2026-04-29

**Authors:** Yoshiko Tojo

**Affiliations:** Department of Radiological Sciences, Ibaraki Prefectural University of Health Sciences, Ibaraki, Ami, Japan

**Keywords:** anterior insula, functional MRI, J-pop music, memory performance, salience network, visual association cortex

## Abstract

Listening to music while studying is common, yet it remains unclear whether self-selected popular music facilitates or interferes with memory performance. This exploratory pilot fMRI study examined whether preferred Japanese pop music (J-Pop), compared with unpleasant sounds and a no-sound baseline, was descriptively associated with differences in visual memory retrieval, subjective focus, and regional brain activity in healthy young adults. Five participants completed a visual encoding and retrieval task under three auditory conditions during fMRI. Behavioral performance, focus ratings, and region-of-interest (ROI) activity in the anterior insula and temporo-occipital visual association cortex were examined using descriptive statistics and nonparametric analyses. Descriptively, recall accuracy and subjective focus were highest in the preferred J-Pop condition, intermediate in the no-sound condition, and lowest in the unpleasant condition; however, these differences did not reach statistical significance. Exploratory ROI analyses showed that anterior insula activity was descriptively higher during the J-Pop condition, whereas temporo-occipital visual association cortex activity was descriptively highest during unpleasant sounds, intermediate during J-Pop, and lowest during the no-sound condition. Brain-behavior correlations were also exploratory and should be interpreted cautiously given the very small sample size. These findings do not establish that preferred J-Pop improves memory performance or supports a specific neural mechanism. Rather, they suggest that this paradigm is feasible and may be useful for generating hypotheses about how affectively positive, self-selected music relates to visual memory retrieval, subjective focus, and salience-related neural processing in future adequately powered studies.

## Introduction

Listening to music while studying is a common everyday practice among students. In both popular media and scientific discourse, classical music has long been highlighted as beneficial for learning and memory, and this idea has often been popularized as a “classical music effect” or the “Mozart effect” (Rauscher et al., 1993). However, evidence for broad cognitive benefits of classical music is mixed, and meta-analytic work suggests that reported effects are small and highly sensitive to experimental context (Pietschnig et al., 2010). Thus, the ecological relevance of classical music as a universal study aid for contemporary students remains questionable.

In actual daily life, only a small minority of university students habitually listen to classical music while studying. Instead, many prefer contemporary popular music—in the Japanese context, this typically means J-Pop. J-Pop songs usually have lyrics in the listener’s native language, and students often find themselves humming or silently singing along. Because meaningful speech and “irrelevant” sounds can disrupt verbal processing and learning, music with lyrics is often assumed to interfere with language-based tasks such as reading or memorizing verbal material (Salamé and Baddeley, 1989; Moreno and Mayer, 2000; Thompson et al., 2012; Lehmann and Seufert, 2017). Individual differences may further modulate such distraction effects, as background music can differentially impact performance depending on factors such as personality (Furnham and Bradley, 1997). From this perspective, listening to one’s favorite J-Pop while studying might be regarded as a distraction rather than a helpful aid.

On the other hand, there are also reasons to expect beneficial or at least non-detrimental effects of preferred popular music in some contexts. Many students anecdotally report that listening to their favorite songs improves mood, reduces stress, and increases motivation to study, and theoretical accounts emphasize that background music effects depend on factors such as arousal, affect, and task demands (Lehmann and Seufert, 2017). Moreover, not all learning domains are equally susceptible to interference. For example, studies focusing on verbal learning have often found little or no benefit of background music (Jäncke and Sandmann, 2010), whereas the impact of music on attention and performance can vary with the emotional and acoustic properties of the music and the cognitive requirements of the task (Fernandez et al., 2019). Thus, there is a clear tension between two intuitions: lyrics and familiarity may draw attention away from the task, while positive affect and engagement may support attention and memory under some conditions.

Furthermore, neuroimaging studies have less frequently examined how Japanese popular music, as it is actually used by students in their daily study routines, modulates brain activity during memory tasks. Existing work on music-related cognition and memory highlights that music can engage distributed brain systems that overlap with general memory and attention networks (Ren and Brown, 2024), but much less is known about whether personally preferred J-Pop engages similar or different mechanisms during visual memory retrieval. In particular, it remains unclear whether listening to one’s favorite J-Pop truly disrupts learning, or whether it might instead support efficient memory processing through salience/engagement mechanisms distinct from those typically emphasized in traditional “classical music” narratives (Lehmann and Seufert, 2017).

The present study addressed this issue by examining how self-selected J-Pop music affects memory and brain activity in comparison with a no-sound condition and unpleasant sounds. Healthy young adults performed a visual picture-based recall task under these three auditory contexts while we measured functional brain responses using fMRI. Behaviorally, we assessed memory performance and subjective ratings of focus. Neurally, we focused on regions implicated in affective and cognitive processing, including the anterior insula as a key area for evaluating pleasantness and salience and a central node of the salience network (Seeley et al., 2007; Sridharan et al., 2008; Menon and Uddin, 2010) and temporo–occipital visual association cortices involved in higher-order visual and memory-related processing. By contrasting a personally meaningful musical context (self-selected J-Pop) with a neutral baseline and an aversive auditory context, we aimed to clarify whether the music that students actually want to listen to truly hinders or supports learning, and how any such effects are reflected in the underlying neural networks.

## Methods

### Participants

Six healthy right-handed university students initially participated in the study. One participant was excluded from all analyses due to incomplete fMRI data, leaving a final sample of five participants for the final behavioral and fMRI analyses (2 women, 3 men; mean age = 21.5 years, range = 21–22). All participants had normal or corrected-to-normal vision and reported no history of neurological or psychiatric disorders. None were professional musicians; however, most reported regularly listening to Japanese popular music (J-Pop) in their daily lives. All participants provided written informed consent prior to participation.

### Visual and auditory stimuli

**Figure 1:**
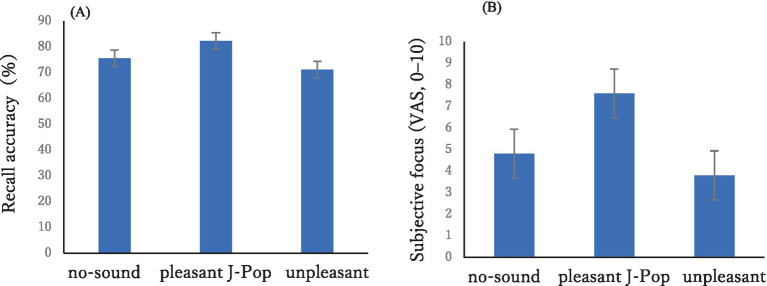
Behavioral performance across auditory conditions. **(A)** Recall accuracy (%) for the no-sound, pleasant J-Pop, and unpleasant conditions. **(B)** Subjective focus ratings on a 0–10 visual analog scale. Bars indicate group means; error bars represent standard errors of the mean (SEM; *n* = 5).

Visual stimuli for the memory task consisted of 18 color pictures of emotionally neutral objects, such as animals and food items, selected to minimize affective bias (Figure 1). The pictures were arranged into six slides, each containing three randomly selected images. These images were later used for the encoding (記銘) and retrieval (想起) phases within each condition. Different image sets were used for each auditory condition; no image was repeated across the three conditions.

Auditory stimulation was manipulated in three conditions: an unpleasant condition, a pleasant J-Pop condition, and a no-sound condition. For the pleasant J-Pop condition, each participant first completed a brief preference screening and then listened to several candidate musical excerpts; the piece they reported as most pleasant and comfortable was adopted as their individual J-Pop stimulus. For the unpleasant condition, we used a composite sound consisting of an earthquake early warning alarm and ambulance sirens, which had been confirmed in a pilot survey to be strongly aversive. In the no-sound condition, only the scanner noise was present (no-sound condition baseline), with no additional auditory stimulation. In all conditions, participants were also exposed to the continuous background noise of the MRI scanner; the three auditory contexts differed only in the presence and type of additional sounds.

Pleasantness/unpleasantness ratings of the auditory context were obtained separately for all three auditory conditions: pleasant J-Pop, unpleasant sounds, and the no-sound condition (scanner noise only). These ratings referred to the auditory context during task performance, not to the visual pictures themselves. The visual stimuli were evaluated separately to confirm their affective neutrality.

For each auditory stimulus, participants rated perceived pleasantness/unpleasantness on a 10-cm visual analog scale (VAS). The VAS was administered using a paper-and-pencil format, and ratings were obtained for all three conditions, including the no-sound condition (scanner noise). The left anchor was labeled 「耐え難いストレスを感じている」 (“experiencing unbearable stress”), the right anchor 「最高に快適である」 (“extremely pleasant”), and the center 「何も感じていない」 (“neither pleasant nor unpleasant”).

Marks on the line were first converted to a scale ranging from −5 to +5, with 0 representing the neutral midpoint, positive values indicating greater pleasantness, and negative values indicating greater unpleasantness. For descriptive purposes, we also expressed responses as the absolute distance from the neutral point on a 0–10 scale, such that higher values reflected stronger affective intensity regardless of valence.

Subjective focus rating. In addition to pleasantness/unpleasantness, participants rated how well they could maintain attention during the task in each auditory condition using a paper-and-pencil 10-cm VAS anchored from “not focused at all” to “extremely focused.” Focus ratings were obtained for all three conditions, including the no-sound condition (scanner noise).

### Memory task and experimental design

The memory task consisted of a visual encoding phase (記銘課題) and a visual retrieval phase (想起課題), performed under the three auditory conditions (pleasant J-Pop, unpleasant sounds, no-sound condition) in a within-subjects design.

During the encoding phase, participants were presented with six slides, each containing three of the neutral pictures (18 images in total). Each slide was displayed for 20 s. Participants were instructed to carefully view the images and memorize them for a later memory test, paying attention both to which objects appeared and to their order.

During the retrieval phase, participants performed a true/false judgment task about the previously presented images. A series of nine statements referring to the encoding images was presented, each for 20 s. The statements probed either the presence, the frequency/category occurrence, or the ordinal position of specific items. For example, one question was “りんごは6番目の画像にあった (The apple appeared as the sixth image).” Another example was “果物が2回出てきた (A fruit appeared twice).” Participants were instructed to judge whether each statement was true or false based on their memory of the encoding phase. If they believed the statement was true, they signaled this by lightly squeezing a hand-held button; the button press was recorded by the system and produced a confirmation buzzer audible only to the operator in the control room (outside the scanner room), not to the participant. If they believed the statement was false, they were instructed to do nothing. No button press was treated as a “false” response (go/no-go true/false format), which was adopted to avoid speech during scanning and minimize head motion. Thus, each trial required a true/false judgment based on visual episodic memory. Different retrieval statements were used for each auditory condition; no statement was repeated across the three conditions.

Across the experiment, the encoding and retrieval trials were presented under each of the three auditory conditions. The timing and number of blocks were identical across conditions and participants, and the order of auditory conditions was pseudo-randomized across participants to minimize order effects.

### MRI data acquisition

Imaging data were acquired on a 3.0 T MAGNETOM Lumina (Siemens Healthcare, Erlangen, Germany) MRI scanner equipped with a 32-channel head coil at Ibaraki Prefectural University of Health Sciences. Functional images sensitive to blood oxygenation level–dependent (BOLD) contrast were obtained using a T2*-weighted gradient-echo echo-planar imaging (EPI) sequence with the following parameters: repetition time (TR) = 2000 ms, echo time (TE) = 35 ms, flip angle = 90°, field of view (FOV) = 240 × 240 mm^2^, matrix size = 256 × 256, slice thickness = 3.0 mm, no interslice gap, and 36 axial slices covering the whole brain. Each functional run consisted of 90 volumes.

High-resolution T1-weighted structural images were acquired for anatomical reference using a MPRAGE sequence (TR = 1,300 ms, TE = 2.88 ms, inversion time (TI) = 900 ms, flip angle = 90°, FOV = 256 × 256 mm^2^, matrix = 256 × 256, slice thickness = 3.0 mm).

### fMRI preprocessing

Functional data were preprocessed using SPM12 (Wellcome Center for Human Neuroimaging, London, United Kingdom) implemented in MATLAB (R2025a, MathWorks, Natick, MA). Note that Siemens scanners automatically acquire several dummy volumes (before the first saved volume) to allow for magnetic field stabilization. Because these dummy volumes are not included in the stored fMRI data, no initial volumes needed to be discarded during preprocessing.

Each participant’s structural T1-weighted image was co-registered to the mean functional image and segmented into gray matter, white matter, and cerebrospinal fluid. The resulting normalization parameters were applied to the functional images to spatially normalize them to the standard MNI template, resampled to 3 × 3 × 3 mm^3^ voxels (Friston et al., 1995). Finally, the normalized functional images were spatially smoothed with an isotropic Gaussian kernel of 8 mm full width at half maximum (FWHM).

### First-level statistical analysis

At the individual participant level, task-related BOLD responses were modeled using a general linear model (GLM) in SPM12 (Friston et al., 1994). Separate regressors were created for each auditory condition (pleasant J-Pop, unpleasant sounds, and no-sound condition), modeling the retrieval blocks as boxcar functions with the duration of each block and convolved with the canonical hemodynamic response function. Six head motion parameters were included as nuisance regressors to reduce movement-related artifacts (Friston et al., 1996). A high-pass filter with a cutoff period of 128 s was applied to remove low-frequency drifts.

For each participant, contrast images were then generated to compare auditory conditions during retrieval, including pleasant J-Pop > no-sound condition, pleasant J-Pop > unpleasant sounds, and unpleasant sounds > no-sound condition.

### Second-level and ROI analyses

The resulting individual contrast images were then carried forward to the group-analysis stage. At the group level, these contrast images were entered into random-effects analyses to provide a descriptive summary of condition-related activation patterns across participants.

ROI analyses were then conducted as exploratory summaries focusing on brain areas previously implicated in affective and memory-related processing. Specifically, we examined the anterior insula (aINS), which has been implicated in salience-related and affective processing, and temporo–occipital visual association cortices, including lateral occipital and fusiform regions, implicated in higher-order visual and memory-related processing. ROIs were defined bilaterally using the Automated Anatomical Labeling (AAL) atlas implemented in the WFU PickAtlas toolbox (Tzourio-Mazoyer et al., 2002; Maldjian et al., 2003).

For each participant, mean T values were extracted from each ROI for the relevant contrast maps. Here, mean T values refer to the average contrast-related T statistics across voxels within each ROI and were used as exploratory ROI summary measures. These ROI summary values were then summarized descriptively across conditions and analyzed using nonparametric tests because of the very small sample size. In addition, exploratory brain–behavior relationships between ROI summary values, recall accuracy, and subjective focus were examined using Spearman rank correlations.

### Behavioral data analysis

Behavioral analyses were performed in Python using SciPy (Virtanen et al., 2020) and pandas (McKinney, 2010). For each participant and condition, we computed recall performance (percentage of correctly answered true/false statements about the pictures) and mean VAS ratings of (i) pleasantness/unpleasantness and (ii) subjective focus. Because of the small sample size and non-normal distributions, these measures were analyzed using non-parametric tests. Condition effects were first examined with Friedman tests, followed by Wilcoxon signed-rank tests for pairwise comparisons. Holm’s method was used to adjust *p* values for multiple comparisons (Holm, 1979).

### Ethics statement

Ethical approval for this study was obtained from the Ethics Committee of Ibaraki Prefectural University of Health Sciences (approval e519). All procedures were conducted in accordance with the Declaration of Helsinki. All participants provided written informed consent prior to participation and received an explanation that they could withdraw from the study at any time without penalty.

## Behavioral results

### Recall performance

Mean recall performance for each auditory condition is shown in Figure 1A. Participants showed the highest accuracy in the pleasant J-Pop condition (M = 82.2%, SEM = 3.0), followed by the no-sound condition (M = 75.6%, SEM = 1.6), with the lowest accuracy in the unpleasant condition (M = 71.1%, SEM = 1.8).

A Friedman test was conducted to compare recall accuracy across the three auditory conditions (no-sound, pleasant J-Pop, unpleasant). The test did not reach statistical significance, χ^2^(2) = 4.60, *p* = 0.10. Although not significant, the descriptive pattern matched that shown in Figure 1A, with the highest accuracy in the pleasant J-Pop condition, followed by the no-sound condition and the lowest accuracy in the unpleasant condition. Follow-up Wilcoxon signed-rank tests revealed no significant pairwise differences after Holm correction (all adjusted *p* values = 1.00), but the same directional pattern was observed in all comparisons.

### Subjective focus

Figure 1B summarizes subjective focus ratings on a 0–10 visual analog scale. Focus ratings were highest in the pleasant J-Pop condition (M = 7.6, SEM = 0.83), intermediate in the no-sound condition (M = 4.8, SEM = 0.77), and lowest in the unpleasant condition (M = 3.8, SEM = 0.77).

A Friedman test was also conducted on subjective focus ratings. The test did not reach statistical significance, χ^2^(2) = 3.20, *p* = 0.20. Nevertheless, the descriptive pattern paralleled the accuracy results and those depicted in Figure 1B, with the highest focus in the pleasant J-Pop condition, intermediate focus in the no-sound condition, and the lowest focus in the unpleasant condition. Follow-up Wilcoxon signed-rank tests showed no significant pairwise differences after Holm correction (all adjusted *p* values = 1.00), but the same directional trend was observed across comparisons.

### Manipulation check: pleasantness of stimuli

On the original −5 to +5 scale (0 = neutral, positive values indicating pleasantness and negative values indicating unpleasantness), J-Pop was rated as clearly pleasant (M = +3.6, SD = 1.0), unpleasant sounds as clearly aversive (M = −2.8, SD = 0.7), and the pictures as comparatively neutral (M = +0.2, SD = 0.4). When expressed as absolute distance from the neutral point on a 0–10 scale, affective intensity was higher for both J-Pop (M = 8.6, SD = 1.02) and unpleasant sounds (M = 7.8, SD = 0.74) than for the pictures (M = 5.2, SD = 0.40), confirming that the auditory stimuli functioned as clearly pleasant and unpleasant, respectively, while the visual stimuli were experienced as near-neutral.

## fMRI results

### Anterior insula (aINS)

**Figure 2:**
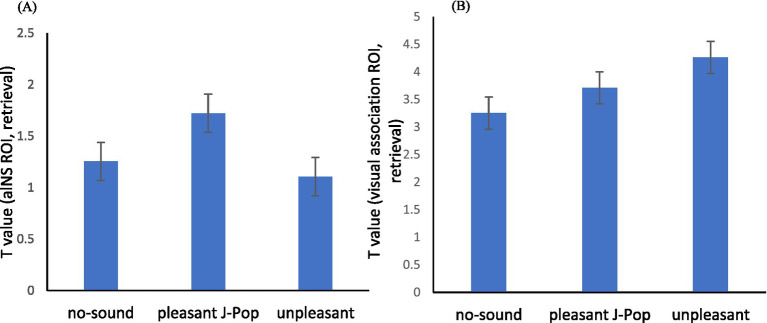
ROI activation during memory retrieval across auditory conditions. **(A)** Mean T values in the anterior insula (aINS). **(B)** Mean T values in temporo–occipital visual association cortices. Bars indicate group means; error bars represent standard errors of the mean (SEM; *n* = 5).

This pattern of anterior insula activation during memory retrieval is illustrated in Figure 2A. ROI analyses showed descriptively higher T values in the J-Pop condition (M = 0.596, SD = 0.097) than in the unpleasant condition (M = 0.528, SD = 0.010). Although these differences did not reach statistical significance in this small sample, the descriptive pattern is presented here as an exploratory ROI summary.

### Temporo–occipital visual association cortex

The corresponding pattern in the temporo–occipital visual association cortex is shown in Figure 2B. Mean T values were descriptively highest in the unpleasant condition (M = 4.27, SD = 3.02), intermediate in the pleasant J-Pop condition (M = 3.72, SD = 2.05), and lowest in the no-sound condition (M = 3.26, SD = 1.21). Thus, in this pilot sample, unpleasant sounds were descriptively associated with higher temporo–occipital ROI summary values than J-Pop or no-sound, whereas J-Pop was associated with intermediate values in this region despite the highest descriptive recall accuracy and subjective focus. A Friedman test conducted on temporo–occipital visual association cortex activation did not reach statistical significance, χ^2^(2) = 1.20, *p* = 0.55. Follow-up Wilcoxon signed-rank tests also did not reveal significant pairwise differences after Holm correction (all adjusted *p* values = 1.00). These findings should therefore be interpreted as descriptive exploratory ROI summaries only.

## Discussion

### Brain–behavior relationships

To examine relationships between behavior and neural activity, Spearman correlations were calculated between recall accuracy, subjective focus, and ROI activation values. Although none of the correlations reached statistical significance (all p values > 0.10), the directional patterns were broadly consistent with the behavioral and ROI results in this pilot sample. Higher recall accuracy and greater subjective focus tended to be associated with greater anterior insula activation, whereas associations with temporo–occipital visual association ROI activity were weaker and did not show a clear performance-linked increase. Given the small sample size, these trends should be interpreted cautiously. They may be compatible with the possibility that pleasant music–related salience or engagement processes are associated with task performance without a proportional increase in posterior visual association ROI summary values, although this interpretation remains exploratory and hypothesis-generating only (Menon and Uddin, 2010; Sridharan et al., 2008).

In this pilot sample, preferred J-Pop was descriptively associated with the highest recall accuracy and subjective focus ratings, whereas unpleasant sounds were associated with the lowest values on these measures. At the neural level, temporo–occipital visual association ROI activity showed the opposite descriptive pattern, with the highest mean T values in the unpleasant condition, intermediate values in the J-Pop condition, and the lowest values in the no-sound condition. Although none of these effects reached statistical significance, this pattern is compatible with the possibility that, under some affectively positive listening contexts, better task performance may occur without a proportional increase in posterior visual association ROI activity.

One possible exploratory interpretation is that unpleasant or less supportive auditory contexts may have been associated with greater perceptual or associative processing demands during retrieval, reflected in descriptively higher activation of temporo–occipital visual association cortices. In contrast, during self-selected J-Pop listening, participants showed descriptively high recall performance and focus with posterior visual association ROI activity that was lower than in the unpleasant condition but higher than in the no-sound condition. Given the very small sample and non-significant effects, however, this interpretation should be treated as hypothesis-generating only.

Our data also point descriptively to a possible role of positive affect and salience-related processing, particularly in the anterior insula, during memory retrieval in this pilot sample. The J-Pop condition was descriptively associated with higher anterior insula activation than both the unpleasant and no-sound conditions. The anterior insula is widely implicated in evaluating subjective salience and affective value, integrating interoceptive and task-related signals, and coordinating interactions among large-scale networks that support goal-directed behavior (Seeley et al., 2007; Sridharan et al., 2008; Menon and Uddin, 2010). In this context, descriptively higher activation in this region during J-Pop recall may be compatible with the possibility that personally preferred music was experienced as a positively valenced, self-relevant context associated with sustained attention during the task. More broadly, this interpretation is compatible with evidence that background music can modulate attentional control depending on its affective properties (Fernandez et al., 2019).

At the same time, anterior insula activation in this study should not be interpreted as specific to mnemonic processing alone. Given the small sample size and the robust affective manipulation, it is also plausible that this response partly reflects reward- and pleasantness-related processing in addition to salience and task engagement.

Importantly, the behavioral correlation structure in the J-Pop condition was descriptively consistent with this interpretation. Across participants, recall performance and subjective focus tended to remain positively related, whereas temporo–occipital visual association ROI activity showed only a weak relationship with performance. In contrast, anterior insula engagement was descriptively higher during J-Pop recall (Figure 2A). Taken together, these exploratory patterns are compatible with the possibility that recall in the J-Pop context may have been associated, in part, with salience- and affect-related engagement rather than with clear performance-linked increases in posterior visual association ROI activity. However, these observations remain tentative and should be interpreted cautiously in light of the non-significant effects and the very small sample size.

Taken together, the present findings should be interpreted as exploratory and hypothesis-generating only. In this very small pilot sample, preferred J-Pop was descriptively associated with higher recall accuracy, greater subjective focus, and increased anterior insula activity, but none of the main behavioral or neural condition effects reached statistical significance. Accordingly, these data do not establish that preferred music enhances memory performance or that anterior insula activity reflects a specific mnemonic mechanism. Rather, they suggest that this experimental paradigm is feasible and may help guide future adequately powered studies examining how affective auditory context relates to visual memory retrieval, subjective focus, and salience-related neural processing.

From an applied perspective, the present results also suggest that self-selected, emotionally positive background music should not automatically be assumed to be detrimental to all forms of studying. At the same time, the current data are far too limited to support broad recommendations about studying with music, and they do not imply that any music, at any volume, would be helpful across tasks or individuals. Instead, the present study is best understood as a feasibility-oriented pilot report indicating that the interaction between preferred music, affective state, and visual memory retrieval is a worthwhile target for future, larger-scale investigation.

## Limitations and future directions

Several limitations of this study should be acknowledged. A particularly important limitation is the extremely small final sample size (*n* = 5), which substantially limits statistical power and precludes strong generalization. Consistent with this limitation, none of the behavioral or neural effects reached conventional levels of statistical significance. In addition, different visual stimulus sets and retrieval statements were used across auditory conditions. Although this approach reduced direct item repetition across conditions, it also introduces a potential confound because auditory-condition effects cannot be fully separated from stimulus-set differences. The order of auditory conditions was pseudo-randomized across participants to reduce potential order effects and proactive/anterograde interference, but such effects cannot be fully excluded in a sample of this size. The present findings should therefore be regarded as preliminary and hypothesis-generating rather than conclusive. Second, we examined only a single type of task (visual picture-based memory) and one genre of preferred music (self-selected J-Pop) in healthy young adults. The extent to which similar salience-based mechanisms would be observed for other kinds of learning (e.g., verbal or conceptual material), other musical genres, or different age groups remains an open question. Future studies with larger and more diverse samples will be needed to test the generality of these effects and to clarify individual differences in when and for whom studying with music is beneficial.

## Data Availability

The original contributions presented in the study are included in the article/Supplementary material, further inquiries can be directed to the corresponding author.
