# Supplementary material for: Preferred J-pop music and visual memory retrieval: an exploratory pilot fMRI study
Source: Front Hum Neurosci. 2026 Apr 29;20:1775292. doi: 10.3389/fnhum.2026.1775292 (PMC13168035; doi:10.3389/fnhum.2026.1775292)
Supplement: Supplementary file 1 [file Table_1.docx]

**Supplementary Tables**

*Preferred J-Pop Music and Visual Memory Retrieval: An Exploratory Pilot fMRI Study*

**Supplementary Table S1. Participant-level behavioral values across auditory conditions.**

*Recall accuracy is expressed as the percentage of correctly answered retrieval statements. Subjective focus was rated on a 0–10 visual analogue scale. Higher values indicate greater subjective focus.*

| **Participant** | **Recall accuracy (%) No-sound** | **Recall accuracy (%) Pleasant J-Pop** | **Recall accuracy (%) Unpleasant** | **Subjective focus（VAS,0-10） No-sound** | **Subjective focus（VAS,0-10） Pleasant J-Pop** | **Subjective focus（VAS,0-10） Unpleasant** |
| --- | --- | --- | --- | --- | --- | --- |
| **P1** | 77.8 | 88.9 | 66.7 | 5 | 9 | 4 |
| **P2** | 88.9 | 88.9 | 55.6 | 3 | 10 | 2 |
| **P3** | 77.8 | 77.8 | 77.8 | 4 | 6 | 3 |
| **P4** | 66.7 | 77.8 | 100 | 4 | 5 | 3 |
| **P5** | 66.7 | 77.8 | 55.6 | 8 | 8 | 7 |

**Supplementary Table S2. Participant-level temporo–occipital ROI summary values across auditory conditions.**

*Values represent the average contrast-related T statistics across voxels within the temporo–occipital ROI and are provided as exploratory descriptive summaries.
*NA, not available. For Participant 1, the temporo–occipital ROI summary value in the no-sound condition was unavailable.*

| **Participant** | **Temporo–occipital mean T No-sound** | **Temporo–occipital mean T Pleasant J-Pop** | **Temporo–occipital mean T Unpleasant** |
| --- | --- | --- | --- |
| **P1** | NA | 7.76 | 9.98 |
| **P2** | 3.07 | 2.64 | 2.80 |
| **P3** | 4.79 | 3.07 | 4.18 |
| **P4** | 3.72 | 3.00 | 3.21 |
| **P5** | 1.45 | 2.13 | 1.17 |
